# Long noncoding RNAs (lncRNAs) dynamics evidence immunomodulation during ISAV-Infected Atlantic salmon (*Salmo salar*)

**DOI:** 10.1038/srep22698

**Published:** 2016-03-04

**Authors:** Sebastian Boltaña, Diego Valenzuela-Miranda, Andrea Aguilar, Simon Mackenzie, Cristian Gallardo-Escárate

**Affiliations:** 1 Laboratory of Biotechnology and Aquatic Genomics, Interdisciplinary Center for Aquaculture Research (INCAR), University of Concepción, Concepción, Chile; 2 Institute of Aquaculture, University of Stirling, Stirling, UK

## Abstract

Despite evidence for participation in the host response to infection, the roles of many long non-coding RNAs (lncRNAs) remain unknown. Therefore, the aims of this study were to identify lncRNAs in Atlantic salmon (*Salmo salar*) and evaluate their transcriptomic regulation during ISA virus (ISAV) infection, an Orthomyxoviridae virus associated with high mortalities in salmonid aquaculture. Using next-generation sequencing, whole-transcriptome analysis of the *Salmo salar* response to ISAV infection was performed, identifying 5,636 putative lncRNAs with a mean length of 695 base pairs. The transcriptional modulation evidenced a similar number of differentially expressed lncRNAs in the gills (3,294), head-kidney (3,275), and liver (3,325) over the course of the infection. Moreover, analysis of a subset of these lncRNAs showed the following: (i) Most were similarly regulated in response to ISA virus infection; (ii) The transcript subsets were uniquely modulated in each tissue (gills, liver, and head-kidney); and (iii) A subset of lncRNAs were upregulated for each tissue and time analysed, indicating potential markers for ISAV infection. These findings represent the first discovery of widespread differential expression of lncRNAs in response to virus infection in non-model species, suggesting that lncRNAs could be involved in regulating the host response during ISAV infection.

Large-scale transcriptomic studies have led to surprising discoveries, including that <10% of the mammalian genome is dedicated to protein coding and that the genome contains a vast amount of non-protein coding transcripts, which has resulted in debate about the role of non-coding RNAs (ncRNAs) in cell biology^1^,^2^. In contrast to protein coding genes, it is possible that the non-coding portion of the genome is related to organism complexity and crucial regulatory processes^3^,^4^. Traditionally, the regulatory functions of RNA were thought limited to roles as ribosomal, messenger, and transfer RNAs. However, ncRNAs have been classified as housekeeping RNAs, microRNAs, small interfering RNAs, PIWI-interacting RNAs, small ncRNAs (<200 nucleotides [nt] in length), and long ncRNAs (lncRNAs, >200 nt in length)^5^.

Specifically, lncRNAs are endogenous cellular RNAs that are mRNA-like in length but with an absent or reduced coding potential (open reading frames (ORF) >30 amino acids). These RNAs include tens of thousands of polyadenylated and non-polyadenylated lncRNAs that are anti-sense, intronic, intergenic, and that overlap with protein coding loci^5^. Once thought to be transcriptional noise, lncRNAs have been shown to regulate a variety of biological processes. While the complex paradigm of RNA-based gene regulation is slowly being revealed, there is a growing body of evidence for a central role of lncRNAs in controlling gene regulation^6^,^7^. A recent review estimated that the total number of lncRNAs is likely ~20,000 transcripts, but, to date, only ~200 lncRNAs have been characterized^8^.

Among the most well studied lncRNAs, *Xist* and *Air* epigenetically silence transcription by targeting chromatin-modifying complexes of particular genes in trans and cis isomers, respectively^9^,^10^. Other lncRNAs act at the post-transcriptional level. These include *H19*, which is the precursor of miR-675, a moderator of cell growth^11^; and *MALAT1*, which forms a molecular scaffold for several proteins present in nuclear speckles and which regulates pre-mRNA alternative splicing^12^.

Although infection and disease are major driving forces of survival and, consequently, adaptation, the potential roles of lncRNAs during the defense response remain unknown. Since the functions of lncRNAs are highly pleiotropic, ranging from gene regulation and small ncRNA precursor development^13^ to cell development and cancer growth^14^,^15^, lncRNAs are very likely involved in virus infection. Early studies of the relationship between ncRNAs and viruses mainly focus on micro RNAs^16^,^17^, while the roles of lncRNAs are not well studied. However, there is emerging evidence that cellular lncRNA expression can be regulated by virus infection.

Differentiated lncRNA expression occurs in response to viral infection^18^,^19^. Specifically, transcriptome-wide deep sequencing revealed the differential expression of more than a thousand potential lncRNAs during severe acute respiratory syndrome coronavirus (SARS-CoV) infection in mice^20^, and several of these lncRNAs are similarly regulated during Influenza A Virus (IAV) infection^21^. Moreover, most lncRNAs show comparative regulatory responses between SARS-CoV and Orthomyxoviridae infections, but lncRNAs have kinetic type I interferon receptor and STAT1 expression profiles that are distinct to each infection^22^. These findings suggest a widespread differential expression of lncRNAs in response to virus infection, in addition to the involvement of lncRNAs in regulating the defense response.

Infectious salmon anemia (ISA) is a significant disease affecting farmed Atlantic salmon (*Salmo salar* L.). This disease, listed by the World Organization for Animal Health, is caused by a negative sense, segmented, single-stranded RNA virus of the Orthomyxoviridae family^23^. This virus family historically consists of five genera (*Influenza virus A, B*, and *C*; *Isavirus*; and *Thogotovirus*) that differ in their host ranges and transmission routes^24^. Like other Orthomyxoviridae, ISAV mRNAs have capped, heterogeneous 5′-ends, and, due to a need for capped host nuclear RNAs as primers for mRNA synthesis, synthesis is inhibited by amanitin, a specific inhibitor of cellular RNA polymerase II^25^. The outcome of ISAV infection is determined by a complex interaction between the virus and host, which have been characterized by three, ordered waves of gene expression that produce immediate-early, delayed-early, and late responses, as well as replication of the viral genome^26^,^27^. However, the molecular mechanisms and transcriptional dynamics behind the host response to ISA infection are still unknown. Next-generation sequencing technologies facilitate rapid and cost-effective deep transcriptome studies in non-model species. Despite expanded data, surprisingly little remains known about how lncRNAs function, how many different types of lncRNAs exist, or even if most lncRNAs are biologically significant.

In the present study, we hypothesized that ISAV could regulate the expression of lncRNAs in infected fish, thus regulating the antiviral response. To evaluate this, the transcriptomic responses of the gills, liver, and head-kidney of Atlantic salmon infected with the highly virulent HPR7b-ISAV strain were determined through whole RNA-Seq profiling. The results showed a widespread differentiation of lncRNAs in response to ISAV infection, suggesting that these transcripts are involved in regulating the host response to the virus. Moreover, lncRNAs accounted for approximately 5% of all expressed contig during infection. The results of this study contribute to a better understanding of ISAV pathogenesis and the interplay of lncRNAs with the immune response in Atlantic salmon.

## Results

### Viral challenge and reconstruction of ISAV-infected *S. salar* transcriptome

**Figure 1:**
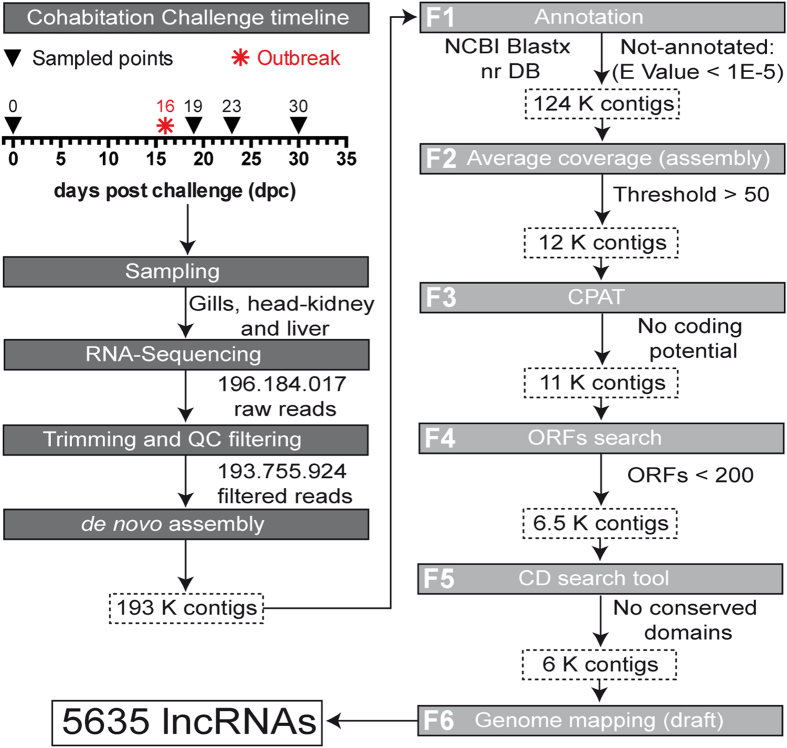
Overview of lncRNA identification process. Outline of computational pipeline and systematic workflow used for discovering specific long non-coding RNAs.

To investigate the regulation of lncRNAs during viral infection, a cohabitation challenge was used to infect *S. salar* with a highly virulent ISAV strain (HPR7b). According to Kaplan-Meier estimates of survival, the different survival rates between trojans and cohabitants were not significant in relation to the lifetime of the fish. Samples were taken at the beginning of the challenge (T0), before any mortalities; at 19 DPC (T1), with the first mortalities; at 23 DPC (T2); and at 30 DPC (T3), when the plateau phase began^26^. Whole-transcriptome analyses were performed on collected gills, liver, and head-kidney tissues using Illumina sequencing. Directional cDNA libraries were constructed by a MiSeq^®^ Illumina sequencer, which profiled polyadenylated, coding, and noncoding transcripts, but not small RNAs^26^,^27^. A large number of reads (193 million) were subjected to quality control filters for the random effects of single slide variability, leaving only transcripts that were either present or marginal in all RNA-Seq experiments. For the filtered reads, *de novo* assembly was performed, and 193,846 contigs were generated with an N50 value equal to 1,171 and effective mapped reads rate of 84.66%. These contigs were used as reference sequences for the identification of putative lncRNAs in *Salmo salar* (Fig. 1). The difficulty to characterize the lncRNA landscape in salmon, lack of a robust lncRNA database and the low genome conservation among the fish consequence of the successive genome duplications in teleost, consequently required a several stage processing of the data with specific objectives. The first was removing the protein-coding transcripts (deleting transcripts that could encode known proteins). As a result, above 70 K contigs were annotated (E-value < 10^−5^) that were discarded from further analysis. Secondly, a highly stringent filter was applied for contig coverage. This filter allows minimizing bias of the *de novo* assembly and discards contigs with low-coverage (12 k contigs with high coverage <50 reads/pb).

As novel protein coding transcripts could associate with novel non-coding transcripts in the prediction process, the Coding Potential Calculator (CPC)^28^,^29^,^30^ was used firstly to evaluate the protein coding potential of novel transcripts and remove putative protein coding transcripts. Secondly we inspected the presence of open reading frames (ORF) >200 and the presence of putative protein conserved domains among all possible translation frames. Based upon the CPC value and the ORF prediction 6 K contigs did not show evidence of coding potential and putative conserved domains within their predicted amino acid sequences. The remaining transcripts were mapped against the latest draft of the *S. salar* genome to ensure their existence at genomic level. Of this set 5,635 did not show partial overlap of predicted loci and represents potential lncRNAs of *S. salar* (Supplementary Table S2). Our integrative analysis of transcriptome data by using computational and highly stringent analytical filters suggests that the transcriptional events detected were largely from ncRNAs and that some could be differentially expressed in response to viral infection.

### Features of salmon tissue lncRNAs

**Figure 2:**
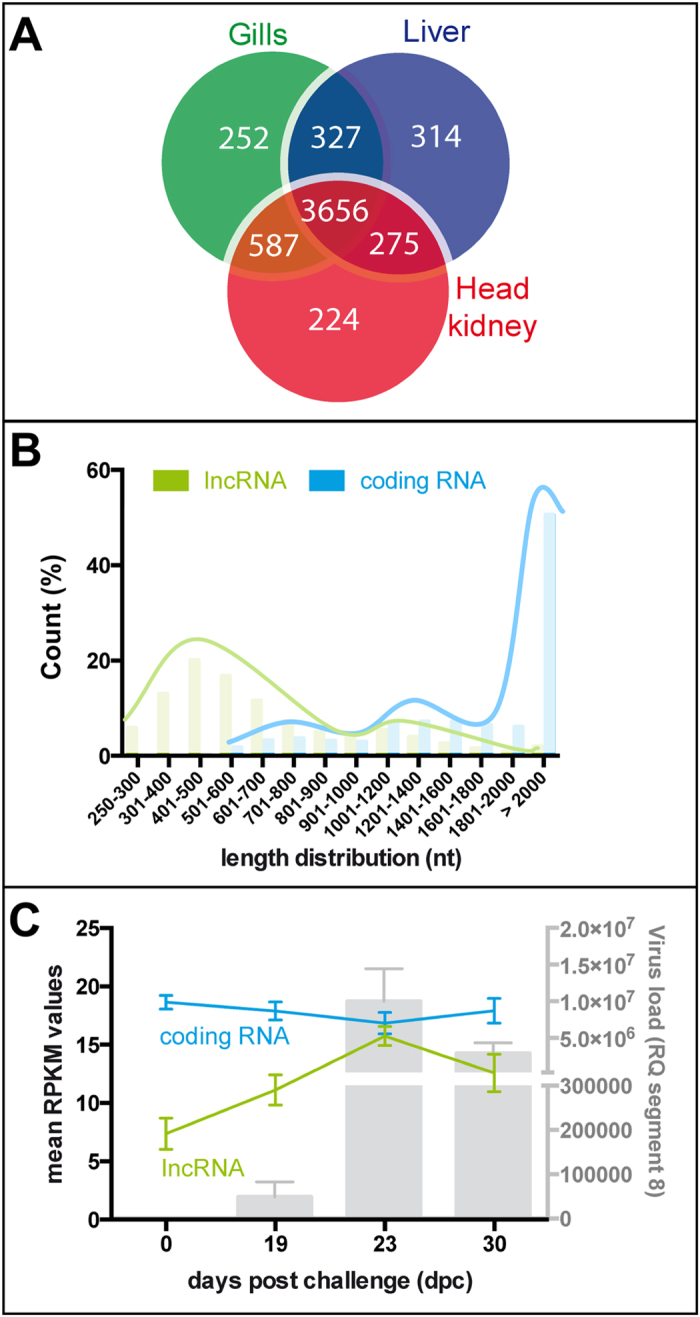
Features of predicted novel lncRNAs. (A) Venn diagram on the distribution of 5,636 putative lncRNAs across three tissues. The overlapping expression profiles of predicted long non-coding RNA transcripts from each tissue are depicted in different colors: gills (Green), liver (Blue), and head-kidney (Red). (B) Length distribution of 5,636 predicted novel lncRNAs and 19,846 coding transcripts. (C) RPKM distribution of maximum expression levels for lncRNAs and mRNAs across the three tissues, based on RNA-Seq data.

Previous studies have shown that lncRNAs are shorter and have significantly lower expression than protein coding transcripts, in addition to being expressed in a tissue-specific manner^31^,^32^. Therefore, the tissue expression of the predicted lncRNAs between sampled tissues was assessed by RNA-Seq. Although most lncRNAs were detectable in all three tissues (3,656), several were tissue-specific numbering 252, 314 and 224 that were exclusively expressed in gills, liver and head kidney respectively (Fig. 2A). On the other hand, the predicted lncRNAs in each tissue were shorter in length (500 nt on average) than coding transcripts (3000 nt on average) (Fig. 2B). Interestingly, the lncRNAs in Atlantic salmon tissues were similar in length to lncRNAs in the mouse and chicken (500–600 nt on average)^31^,^32^.

Another approach for inferring the putative features of long ncRNAs is to examine protein coding genes of interest in correlation with ncRNA expression^29^,^30^. The infection-induced patterns of expression were examined for ncRNAs and protein coding genes. Interestingly, we found that the changes in expression of protein-coding genes (fold changes, Fig. 2C blue line) were significantly associated with the fold changes in expression of the ncRNAs during infection at 23 dpc (*P* values = 7e^−23^ analysis of variance [ANOVA], F test, Fig. 2C, and supplementary material Tables S3 and S4). The expression levels of lncRNAs were 4-fold lower than those of protein coding transcripts at time 0, followed by a slightly increased number of transcripts reaching a maximum at 23 DPC and decrease at 30 DPC (Fig. 2C), which is similar to the expression patterns of lncRNAs in humans and zebrafish under basal conditions^33^,^34^.

**Figure 3:**
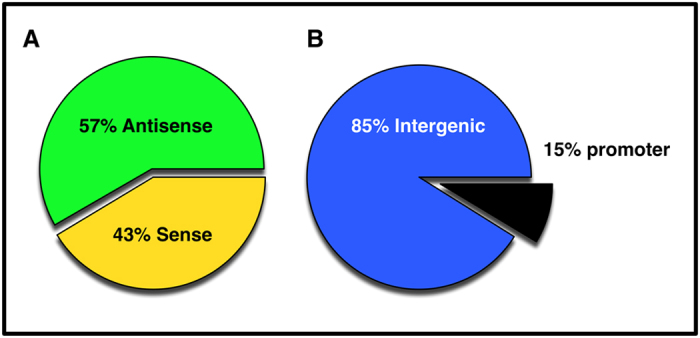
Global classification of lncRNAs. (A) Characteristics of genomic regions differentially expressed during ISAV infection. Unknown genomic regions without any overlapping annotated gene were classified in sense and antisense lncRNAs. (B) Short reads were assigned to one of three nonoverlapping categories. The promoter, intronic, and intergenic categories were defined by the genomic coordinates for known immune related genes and include only reads that map to unique genomic locations.

Further analysis evaluated whether lncRNAs or protein coding transcripts (mRNA) resulted in time-dependent manner in RNA abundance after the ISA virus challenge. At 23 DPC, lncRNA abundance peaked in close correlation with an increase in ISAV load, both of which then decreased until the final sample time. These observations suggest that lncRNAs are expressed in a temporal-specific manner. Meanwhile, mRNAs showed a temporal and tissue-specific modulation, novel lncRNAs expression increased during viral challenge. We observed a large number of reads mapped close to protein coding mRNAs (Fig. 3A,B) in samples from virus-infected fish. From the tissues, we obtained that the lncRNAs mapped to host genomic sites, including many that mapped to nonannotated intergenic regions (Fig. 3B; see Table S6 in the supplemental material). We reasoned that the transcriptional activities detected in nonannotated regions were largely from ncRNAs and that were differentially expressed in response to viral infection.

### Differential expression of lncRNAs during ISAV infection

**Figure 4:**
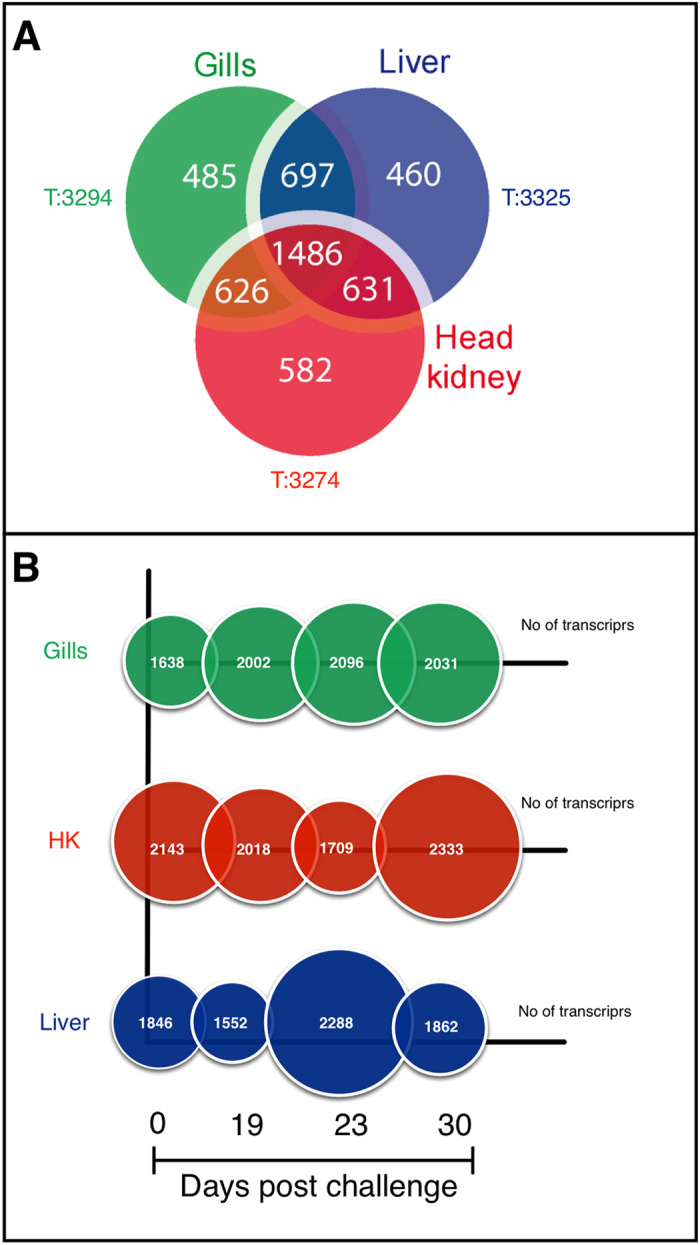
Tissue-wide distribution of lnRNAs. Distribution of 5,636 novel lnRNAs across three tissues. (A) Venn diagram representing the differentially expressed lncRNAs distributed between the gills (Green), liver (Blue), and head-kidney (Red). (B) Distribution of lncRNAs across the different sampling time points.

Of the 5,636 non-overlapping lncRNA loci identified 4,967 were differentially expressed during ISAV infection in each tissue (Fig. 4A). These results indicate a widespread differential regulation of lncRNAs in response to ISAV infection. Furthermore, all of the analyzed tissues and sampling times (T0, T1, T2, T3) were systematically scanned for unannotated regions that encoded for transcripts differentially expressed during viral infection (Fig. 4B).

In total, 1–2 K unannotated RNAs were discovered that did not overlap with any annotated protein coding gene (*S. salar* genome) and that consistently had >2-fold changes in expression during the infection process (Fig. 4B; Supplementary Table S3). For 5,636 of these non-overlapping lncRNA loci, no overlap with any annotated loci was found, indicating that a multiplicity of infection-induced changes in RNA transcript abundance are not detected by conventional RNA-Seq analysis. This result also suggests that other infection-related transcripts llikely remain to be discovered under different experimental conditions.

**Figure 5:**
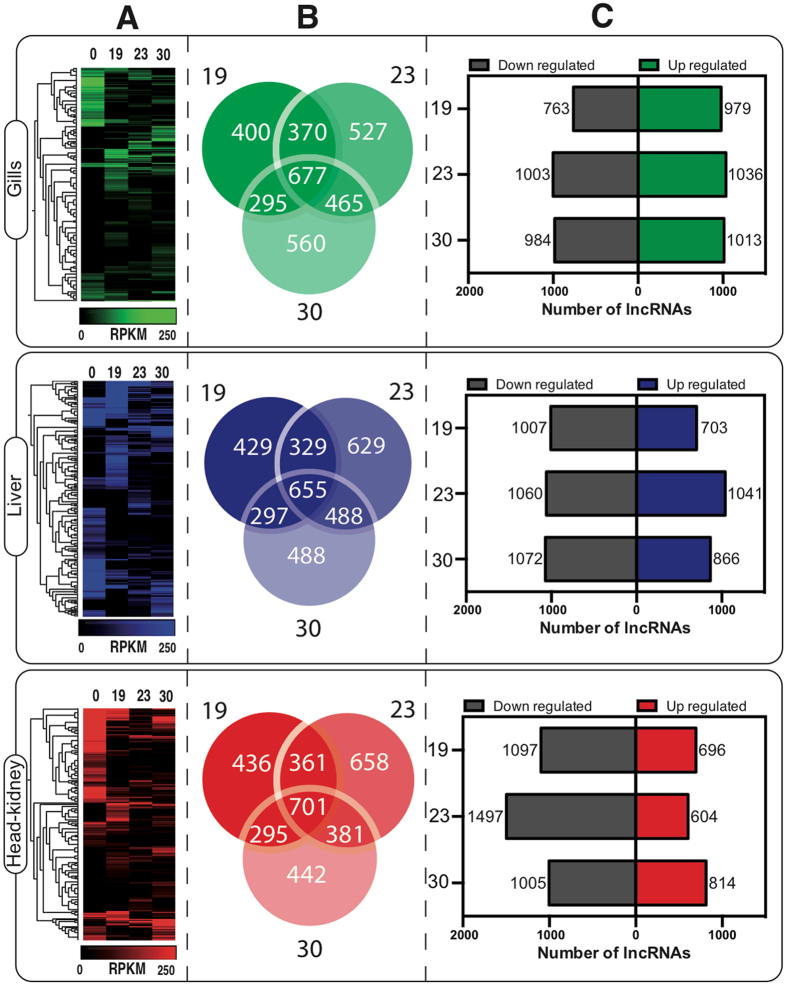
Temporal, specific expression of lncRNAs in the gills. (A) Heat-map and cluster selection of differentially expressed lncRNAs in the gills, liver and headkidney over the course of ISAV infection (green blue and red respectively). Hierarchical clustering with log-transformed RPKM values, showing the most differentially expressed genes (|Fold-change| > 2 and p-value < 0.01) at 19, 23, and 30 DPC. The color intensity represents RPKM values, where colours (green blue and red) indicates a presence and black an absence of regulation. (B) Venn diagram of the differentially expressed lncRNAs at the sampled times. (C) Number of lncRNAs up/downregulated in each tissue infected with ISAV. The bars indicate the fold-change values and RPKM expression values at each sampling point (19, 23, and 30 DPC).

Furthermore, analysis was performed to determine if the levels of lncRNAs and antiviral mRNAs were altered during early or late ISAV infection. Therefore, the expression levels of lncRNAs were evaluated in ISAV-challenged fish at 19, 23, and 30 DPC. The fold-change observed for each tissue at each time point is shown in Fig. 5A–C and Supplementary Figure S1. The results showed a canonical lncRNA gene expression pattern that was triggered by ISAV and regulated differentially in each tissue (gills, liver and head-kidney).

In the gills, ISAV induced a potent increase in upregulated lncRNAs (979 to 1,036 transcripts), with maximum lncRNAabundance at 23 DPC followed by a slight decrease in expression at 30 DPC (Fig. 5A). Overall changes in lncRNA expression in the gills (up/downregulated) increased from 1,742 to 2,039 transcripts at 23 DPC, followed by a slightly decreased number of transcripts (1,997) at 30 DPC. In the head-kidney, the temporal expression of lncRNAs was similar to that observed in the gills, with an initial lower abundance at 19 DPC (1,793) followed by a peak at 23 DPC (2,101) and decrease at 30 DPC (1,819; Fig. 5C). The observed upregulation of head-kidney lncRNAs was lower than that observed in gills, with 696 upregulated lncRNAs at 19 DPC, followed by 604 at 23 DPC and 814 at 30 DPC (Fig. 5C). Finally, ISAV infection induced a potent increase in the expression of lncRNAs in the liver (Fig. 5B), reaching a maximum upregulation at 23 DPC (1,041), followed by a decrease at 30 DPC (866), similar to that observed in the gills.

### Response of lncRNAs to viral infection

RT-qPCR analysis was used to further evaluate the differential expression of a subset of lncRNAs in replicate samples. Seven non-overlapping, differentially expressed lncRNAs and seven protein coding genes known to be regulated during viral infection were selected for the following analyses. The selected lncRNAs displayed significant temporal changes in expression in response to the infection (*P* < 0.05) between at least two consecutive time points. These findings indicate that the differential expression of lncRNAs during ISAV infection are affected by perturbations to antiviral signaling and, importantly, could be associated with an increase efficiency of the immune response to the ISA virus.

**Figure 6:**
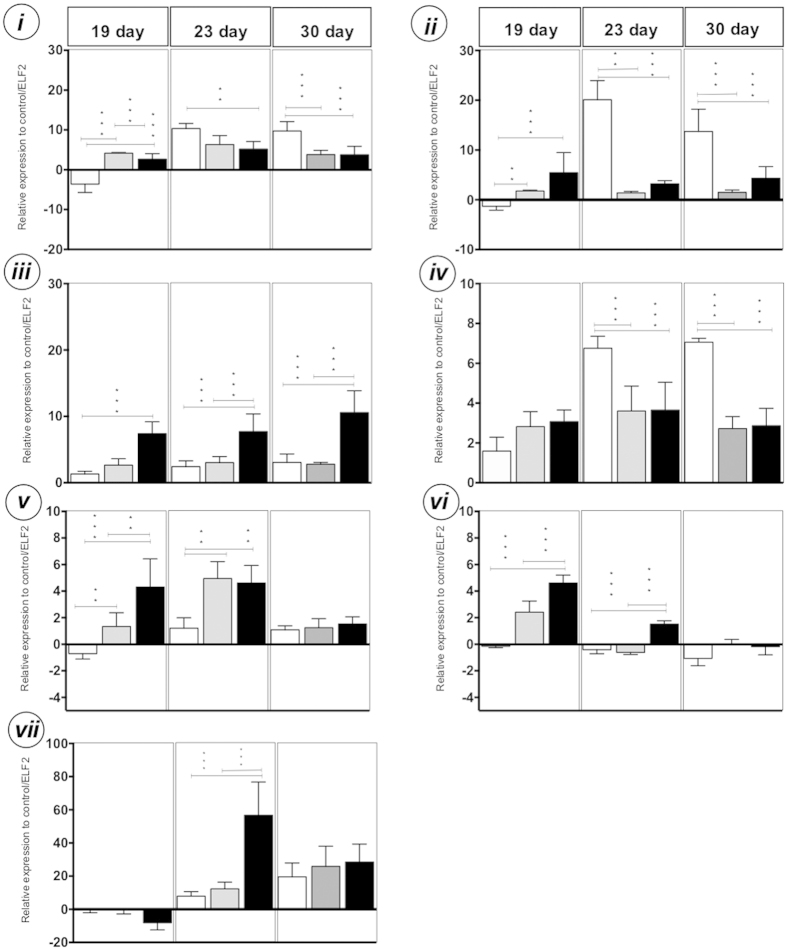
RT-qPCR quantification of candidate lncRNA transcripts. Values represent the maximum relative abundance ratio of mRNA (control: infected) in the gills (white), liver (grey), and head-kidney (black). The lncRNA transcripts investigated in a particular tissue type showed relatively predominant expressions in the specific tissue as compared to other tissues. Values are represented as the mean ± s.d. Two-way ANOVA was performed for (i) Ss_lncRNA_575, (ii) Ss_lncRNA_1421, (iii) Ss_lncRNA_1969, (iv) Ss_lncRNA_2198, (v) Ss_lncRNA_2753, (vii) Ss_lncRNA_4968, and (vii) Ss_lncRNA_4977. Letters represent comparisons (a,b,c) and significance was confirmed through a Bonferroni post-hoc test (*p < 0.05; **p < 0.01; ***p < 0.001).

**Figure 7:**
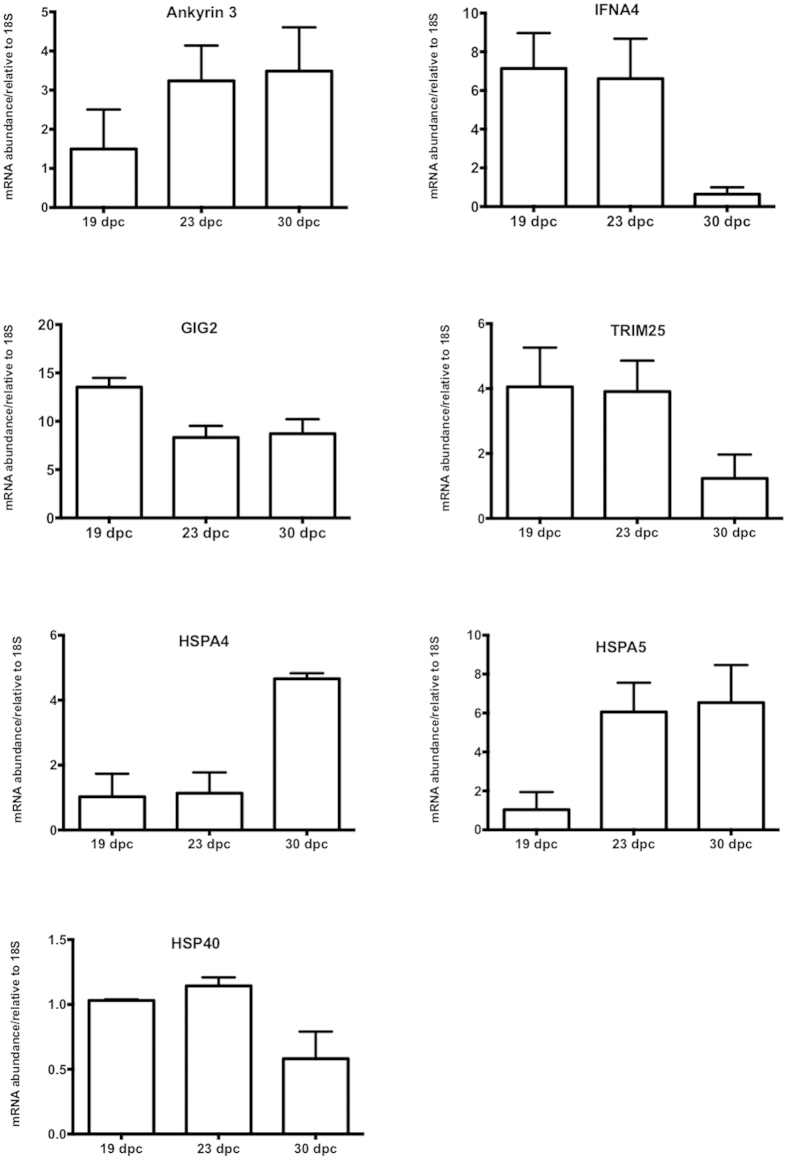
Real-time assay for antiviral mRNAs. RT-qPCR quantification of specific anti-viral mRNA accumulation over a 31 day period post-ISAV challenge. Values shown are the maximum relative abundance ratio of mRNA (control:infected) in ISAV-challenged fish. Values are represented as the mean ± s.d.

Guilt-by-association analysis indicated that Ss_lncRNA_575, Ss_lncRNA_1421, and Ss_lncRNA_4968 lncRNAs could strongly respond to ISAV infections. To study this further, the expression of these lncRNAs was evaluated in fish infected with ISA-HPR7b. All Orthomyxoviridae viruses normally induce a fast lytic infection that initiates cell death 1–2 days post-infection, or at 5–10 days post-infection in the case of the HPR7b ISAV strain (data not shown). Our results show that Ss_lncRNA_4977 expression was regulated by infection at all sampling points (Fig. 6). In contrast, changes in Ss_lncRNA_4968 and Ss_lncRNA_2198 expression were only induced by ISAV during the early stages of infection, but these transcripts were undetectable during later infection stages, when the antiviral response is strongest (Fig. 7). In general, the induction pattern was similar to that measured for *GIG2, Interferon, TRIM25,* and *ankyrin 3* mRNAs.

A strongest increases of the Ss_lncRNA_4977 and Ss_lncRNA_2198 expression were observed in the liver, a tissue in which the ISA virus employs several viral proteins to block the interferon pathway. Increased expression in the antiviral response was also observed for *GIG2* and *IFNA4*, but not for other interferon-stimulated genes such as *HSPA4, HSPA5*, or *HSP40* (Fig. 7). The mostly lncRNAs were significantly upregulated in almost all tissues at 23 and 30 dpi (Fig. 6) and showed a trend similar to antiviral mRNAs during ISAV infection ate the same days. Finally, to validate the differentially expressed genes (fold-changes), the correlation between the relative expression values (RT-qPCR) and the *in silico* approach (RPKM values) was tested. Pearson correlation coefficients evidenced values of 0.94 (Supplementary Table S4).

## Discussion

Constant interactions between viruses and hosts during their co-evolution have shaped the immune system. Previous studies on virus-host interactions have largely focused on protein coding genes. The potential of lncRNAs to regulate important aspects of the cellular machinery during host-pathogen interactions is highlighted by the tightly controlled transcription of lncRNAs and the fact that they appear to play a role in many tissues^35^. Although lncRNAs have been increasingly implicated in infectious disease, only a few have been functionally characterized during the host response. Given that small ncRNAs, such as microRNAs^36^,^37^, have already been shown to have important roles during the host response to infections, it is likely that lncRNAs also play important roles.

Pang *et al*.^19^ showed that lncRNAs have altered expressions during CD8^+^T cell differentiation upon antigen recognition^19^. Additionally, Ahanda *et al*.^38^ identified mRNA-like ncRNAs that were differentially expressed in virus-infected birds^38^, and Josset *et al*.^2^ showed that several lncRNAs were dynamically regulated in mice in response to SARS-CoV and IAV^2^. In regards to fish, most of the literature pertaining to lncRNAs is primarily focused on describing functional roles during ontogenetic development^39^. In the context of identifying novel mRNA-like lncRNAs, this study describes lncRNAs expression from diverse tissues in fish infected with ISAV. Next-generation high throughput sequencing technology was used to detect polyadenylated transcripts, which were then filtered through a computational analysis pipeline to identify putative, novel lncRNAs. To our knowledge, this is the first study using comprehensive deep-sequencing technology that clearly indicates the participation and correlation of lncRNAs in the fish response to viral infection.

As a result, a total of 5,646 putative lncRNAs were identified in Atlantic salmon, where 3,656 evidenced a regulation after viral infection among gills, liver and head-kidney. The reported lncRNAs did not overlap with any other reported lncRNA dataset in fish, due to the unknown degree of lncRNA conservation between taxa and the stringent computational analysis^35^ used in our study. Our custom-designed work-flow filtered out a large portion of transcripts that could represent annotated protein coding transcripts or other non-coding RNA transcripts, such as small ncRNAs. The threshold of each lncRNA gave enrichment results that were highly dependent upon the cutoff used. Due to this, a more robust, threshold-independent method was used. A similar approach has been used in mice under IAV and SARS-CoV infections^2^, demonstrating a finely tuned characterization of ncRNAs.

Among selected features for the identification of lncRNAs, the predicted length of the ORFs has an important role for the identification of lncRNAs. When ORFs cutoff was changed from 300 to 200, the total number of identified lncRNAs varied from 18,854 to 5,635. Therefore, being less strict about the ORF length provides increased bias in the calculation of the lncRNAs which a rise in false-positive rate^40^,^41^. In consequence, 5,635 lncRNAs were identified for *S. salar* (cut-off < 30 amino acids), were classified as novel lncRNAs not previously reported in salmonids. Of these a large portion of lncRNAs was detected in two or more tissues (3,607), meanwhile 1,980 were only present in one tissue (tissue restricted). In agreement with the above mentioned, we hypothesize that the high number of shared transcripts among tissues (64%) also could be related with the role of lncRNAs in the regulation of coding proteins associated to the maintenance of the corresponding tissues.

The expression of individual lncRNA transcripts varied widely in the investigated tissues under viral infection. All three tissues had different subsets of uniquely restricted lncRNA transcripts. This finding is in agreement with previous results on monocytes challenged with bacterial lipopolysaccharide that drives changes in transcriptomic dynamics of lncRNAs suggesting an importance ofthese molecules during the defense response^42^. In this study, the rapid changes observed in lncRNAs during ISAV infection suggests that the abundance of lncRNAs is highly regulated. During the defense response of *S. salar* to ISA infection, a highly selective modulation of immune-like mRNAs is required^26^. Therefore, an intrinsic question is if the turn over rate of lncRNAs is regulated by a mechanism comparable to that regulating the stability of mRNAs during the defense response. Studies of lncRNAs in yeast^43^ have shown that decapping is a crucial mechanism in regulating the stability of mRNAs, where this decapping promotes the rapid and robust induction of genes associated with galactose utilization. Thus, the regulatory mechanism of lncRNA performance may play an essential role in the transcription of mRNA during the defense response to pathogens.

Previous NGS-based studies have also shown that lncRNAs play an important role in other infections with Orthomyxoviridae SARS-Cov and IAV infections^20^,^22^ or during other virus infections such as Japanese encephalitis^44^. Identifying the role of lncRNAs involved in the viral response may be particularly challenging in fish due to the observed genomic diversity between species, including a genome duplication in *Salmo salar*. In our study, as there were no other known variables the differences in the transcriptomic profile of lncRNAs are assumed to be due to ISA virus. This assumption is supported by the variation in transcript number and intensity (Figs 2, 3, 4).

In this study, the most up/downregulated lncRNAs were enriched in genes associated with innate immunity in the gills, liver, and head-kidney. Moreover, lncRNAs had a high tissue specificity, similar to coding genes. This robust correlation has been previously reported in mammals^45^. Alternatively, some of the downregulated lncRNAs might be highly expressed in control tissues and later stages of infection to maintain homeostasis. Viruses are known to hijack host cellular machinery for viral replication and to suppress antiviral responses through a variety of mechanisms. The relevance of host cell factors during ISAV replication has been previously identified, revealing the involvement of several antiviral networks in ISAV replication^26^,^27^. This mechanism has been fully characterized in other Orthomyxoviridae, such as IAV^46^,^47^. As these processes require tight control to ensure successful virus propagation, we propose that lncRNA transcription likely plays a key role in the regulation of gene expression during ISAV infection.

Importantly, the diverse responses of the salmon to ISAV infection made it possible to annotate lncRNA functions in the context of viral infection. HPR7b-ISAV infection triggered a wide range of host defense responses associated with significant differences in the magnitude of the transcriptomic response. As previously shown, HPR7b-ISAV infection is able to promote a strong, tissue-specific, antiviral response in antiviral genes *Mx1, IRFa,* and *Viperin*^26^. The present study sheds new light on lncRNAs that may be involved in host defense to ISAV infection. Specifically, seven lncRNAs were highly correlated with antiviral mRNA expression in all of the examined tissues, none of which have been described before. Among these lncRNAs, Ss_lncRNA_4968, Ss_lncRNA_575, and Ss_lncRNA_1421 were significantly upregulated after HPR7b-ISAV infection. The expression of Ss_lncRNA_2198 was highly correlated with ISAV-segment 7 replication in the liver and was positively correlated with *GIG2*. These results suggest that some lncRNAs might modulate the fish defense response during viral infection, in addition to highlighting the richness of this dataset for future analyses that could generate new hypotheses and understandings on ISA virus pathogenesis and lncRNA functions. To check the sequence conservation of the lncRNAs, we used the synteny block analysis by Ulitsky *et al*.^1^. The limited knowledge of lncRNAs sequence in fish restricted the success of this analysis, which displayed a low conservation across different mammalian species^1^.

The identification of lncRNAs was restricted to polyadenylated transcripts to the detriment of non-polyadenylated lncRNAs^48^,^49^. In addition, this study greatly expanded on the existing annotation of lncRNAs in fish and described significant regulation of 5,636 lncRNAs, most of which have not been previously described following ISA virus infection. Taking into consideration the limitations of the genomic information in *S. salar* (annotation, genomic and chromosome location, DNA scaffolds, etc), we classified the lncRNAs in intronic or promoter region, the results showed that most of the selected lncRNAs were intergenic and, in the case of Ss_lncRNA_4977 and Ss_lncRNA_574, were located tightly linked with innate immune response and antigen presentation gene*s*. However to full characterization of lncRNAs further studies of its molecular basis are required. In agreement with the above in contributing to the general knowledge of lncRNA functions, we expect that this work will aid in experimentally characterising lncRNAs in non-model species. As the function of lncRNAs remains poorly understood in fish, our results become a useful resource for exploring the role of the non-protein coding transcriptome during viral infection. LncRNAs may represent a whole new class of innate immunity signaling molecules^50^, viral-dependent regulators, or even a new layer of gene expression regulation responsible for modulating host responses during viral infection. Similarly, lncRNAs may also represent a new potential class of biomarkers for infectious diseases. The similar differential regulation of lncRNAs in response to ISA virus infection indicates that lncRNA-based signature of virus infection may exist, suggesting additional diagnostic potential for fish infections. In particular, the mechanistic characterization of lncRNAs belonging to the viral response would have broad impacts in fields as fish immunology. Finally, in the near future, it is likely that detailed knowledge of lncRNA regulation and function will be necessary for fully understanding viral pathogenesis in fish.

## Methods

### Ethics statement

Fish were maintained in Aquainnovo facilities in recirculating water at 15 °C under a photoperiod of 12:12 (light:dark) and fed a maintenance diet of commercial pellets once a day. Water quality indicators, such as dissolved oxygen, pH, nitrite, and ammonia, were analyzed periodically, and the measured values were acceptable considering the particular requirements of this species. All animal procedures were carried out under the guidelines of “International Guiding Principles for Biomedical Research Involving Animals” of European Union Council (2010/63/EU) and fulfilling the statements of the Animal Welfare Protocol (AWP) from Aquainnovo. All experimental protocols were approved by the ethical committee of the University of Concepción.

### Biological samples and high-throughput transcriptome sequencing

From a recent study by our group, Illumina sequencing data for *S. salar* challenged with ISAV were obtained from the Sequence Read Archive (Acc. No. SRX658605; http://www.ncbi.nlm.nih.gov/sra)^26^,^37^. Briefly, transcriptome sequencing was conducted in Atlantic salmon (*S. salar*) challenged by cohabitation with the ISA virus. The gills, head-kidney, and liver were sampled from *S. salar* prior to the challenge (day 0) and after the first evidences of ISA outbreak at 19, 23, and 30 days post-challenge (DPC) (Fig. 1). From 30 mg of each tissue, total RNA was isolated using the RiboPure™ Kit (Ambion, USA) according to the manufacturer’s instructions. RNA concentration and purity were estimated using the NanoDrop 1000 Spectrophotometer (Thermo Scientific, USA), while the RNA integrity number was evaluated with a 2200 TapeStation (Agilent Technologies, USA) using R6K screen tape. Samples with RNA integrity number values above eight and with a 260/280 ratio equal to 1.8 were used for library construction. The cDNA libraries were constructed with RNA isolated from each tissue at the different sample times and sequenced in the MiSeq Sequencing Platform (Illumina, USA) in six different runs.

### *De novo* assembly

Sequencing data analysis was performed using the CLC Genomics Workbench software (CLC bio, Denmark). Raw reads were filtered by quality and adapter/index trimmed. CLC bio’s *de novo* assembly algorithm was used to create a contig list from previously filtered reads using a mismatch cost = 2, insertion cost = 3, deletion cost = 3, length fraction = 0.8, similarity fraction = 0.8, and a minimum contig length = 250. Finally, contigs were adjusted by mapped reads, and end gaps were treated as mismatches. Identifying putative long non-coding transcripts relies on removing those sequences that could encode for a protein. For this, different filters were systematically applied for the identification of lncRNAs (Fig. 1). Expression values were estimated as reads per kilobase of exon model per million mapped reads (RPKM) and then normalized to the total number of assembled contigs, using state numbers in reads per 1000000.

### Contig annotation and coverage filter

Contig annotations were performed based on protein similarity. For this, nucleotide sequences were translated to protein sequences and blasted against the non-redundant BLASTx protein database using a word size = 3, gap cost existence = 11, extension = 1, and a BLOSUM62 matrix. A strict E value of 1 × 10^−5^ was used as threshold for the identification of unannotated sequences. Therefore, sequences with higher E-values were treated as unannotated transcripts and used for further analysis. Later, contigs with an average read coverage below 50 were also removed.

### Coding potential and genome mapping filter

Coding potential is a support vector machines-based classifying system that comprehensively scores transcript characteristics, including the presence and integrity of predicted ORFs, similarity to known protein sequences, and conservation of a single frame. The following three approaches were used to identify and discard transcripts that could encode for proteins: (i) The Coding Potential Assessment Tool was used to discriminate coding and noncoding transcripts from a large pool of candidates^28^,^30^; (ii) The transcripts with an ORF length of less than 30 amino acids (defined for lncRNA as <200 base pairs) were chosen; and (iii) An NCBI conserved domain search was used to identify transcripts that could encode for protein-conserved domains. The remaining transcripts were mapped against the latest version of the *S. salar* genome (Acc. No. AGKD00000000.4), considering a mismatch cost = 2, insertion cost = 3, deletion cost = 3, length fraction = 0.8, and a similarity fraction = 0.8. The predicted lncRNAs were aligned against previously obtained datasets for *Salmo salar*. Likewise, the predicted lncRNAs were checked for matches to any protein coding isoforms (BLASTx). Those transcripts that had any match with protein coding isoforms were removed from further analyses.

### *In silico* lncRNAs expression

Different RNA-Seq analyses were performed for each sample and tissue by mapping filtered reads against putative lncRNAs. The considered parameters included a minimum read length fraction = 0.9, minimum read similarity fraction = 0.9, and unspecific read match limit = 10 in relation to the reference dataset. Expression values were estimated as RPKM and normalized against the total set, using state numbers in reads per 1000000. To identify lncRNAs highly regulated during ISAV infection in each tissue, the Z-test was used for statistical analysis^51^. This test counts data and compares single samples against one another. The Z-test is based on an approximation of binominal distribution by using normal distribution and taking proportions rather than raw counts into consideration. Additionally, p-values were false discovery rate-corrected. A Volcano plot was used to select and extract the most differentially expressed transcripts, with highly regulated transcripts being those with |fold changes| > 2 and p-values < 0.01, as compared to the control group (day 0).

### RT-qPCR validation

Quantitative real-time PCR was used to validate the expressions of ncRNAs. Primer sets for SYBR Green quantitative reverse transcription-PCR (RT-qPCR) were designed using Primer3^52^. For each locus of interest, two or more pairs of primers were designed, and the primer with the best amplification efficiency in samples across all tissues was selected for subsequent quantifications. RT-qPCR was performed using the StepOnePlus™ Real-Time PCR System (Applied Biosystems, Life Technologies, USA), and each assay was run in triplicate using the Power SYBR Green PCR Master Mix (Applied Biosystems). Each reaction was conducted with a volume of 10 μL using the Maxima® SYBR Green/ROX qPCR Master Mix (Thermo Scientific, USA). Five putative housekeeping genes (*Elongation factor 2-alpha* (*ELF-2*)*, β-actin, GAPDH, 18* *s* rRNA, and *S20*) were statistically analyzed using the NormFinder algorithm to assess transcriptional expression stability. Through analysis, *ELF-2* was selected for gene normalizations. Relative expression levels were determined by applying the ΔΔCt method, using *ELF-2* as an endogenous control and for gene normalization. Primer sequences are provided in Supplementary Table S1.

## Additional Information

**How to cite this article**: Boltaña, S. *et al*. Long noncoding RNAs (lncRNAs) dynamics evidence immunomodulation during ISAV-Infected Atlantic salmon (*Salmo salar*). *Sci. Rep.* **6**, 22698; doi: 10.1038/srep22698 (2016).

## Supplementary Material

Supplementary Information

Supplementary Dataset 1

Supplementary Dataset 2

Supplementary Dataset 3

Supplementary Dataset 4
