# Supplementary material for: Long noncoding RNAs (lncRNAs) dynamics evidence immunomodulation during ISAV-Infected Atlantic salmon (Salmo salar)
Source: Sci Rep. 2016 Mar 4;6:22698. doi: 10.1038/srep22698 (PMC4778034; doi:10.1038/srep22698)
Supplement: Supplementary Information [file srep22698-s1.pdf]

# Long noncoding RNAs (lncRNAs) dynamics evidence immunomodulation during ISAV-Infected Atlantic salmon (*Salmo salar*)

Sebastian Boltaña[1¶]*, Diego Valenzuela-Miranda[1¶], Andrea Aguilar[1], Simon Mackenzie[2], Cristian Gallardo-Escárate[1]

[1]Laboratory of Biotechnology and Aquatic Genomics, Interdisciplinary Center for Aquaculture Research (INCAR), University of Concepción, Concepción, Chile
[2]Institute of Aquaculture, University of Stirling, Stirling, UK

* Corresponding author
E-mail: sboltana@udec.cl (S.B)
[¶] These authors contributed equally to this work

**Supplementary figure 1**

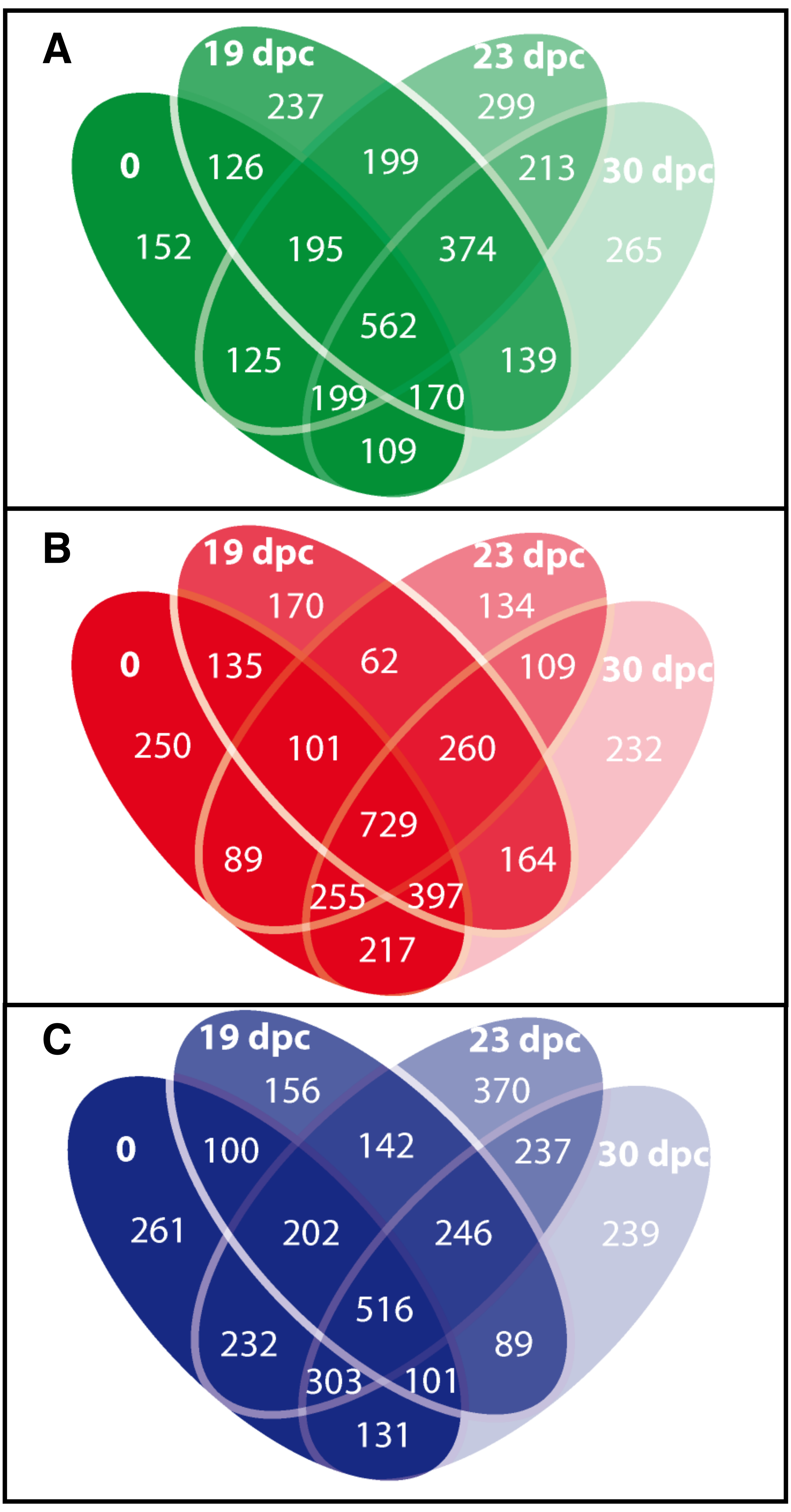


**Supplementary table 1**

| lncRNA | primer sequence (5` - 3`) |
|---|---|
| Ss_lncRNA_575 | FW: GGACCCCTCGTAGCCTAATG<br>RV: TTCATACCCACAGCCACTCT |
| Ss_lncRNA_1421 | FW: CGGTTTGTTAATTAGCCCTCGC<br>RV: CTCTGAGTGGAGTGAAGCCTG |
| Ss_lncRNA_1969 | FW: GGGACAGGGGATGGGTTCTA<br>RV: CGTGCAAGTGTGTGTGCATT |
| Ss_lncRNA_2198 | FW: GCCGTCAGTCACTGAGTGAA<br>RV: ACCACTGTTCTACGCCACAG |
| Ss_lncRNA_2753 | FW: CCGAGGCAGTTTAAGGGACA<br>RV: TGAATCCTTGGGAATGGGCA |
| Ss_lncRNA_4968 | FW: GAGCCAAGGTCTTACGGCTT<br>RV: GGTCTCAGTTCCCAAACGCT |
| Ss_lncRNA_4977 | FW: CAACGCAGACAACCAGAAGC<br>RV: GCTCATCCGAAGCACAAAGC |

**Supplementary table 6**

| long non-coding | Sense | Location | Nearest gene |
|---|---|---|---|
| Ss_lncRNA_574 | Sense | intergenic | E3 ubiquitin-protein ligase HERC3 |
| Ss_lncRNA_1421 | Sense | intergenic | myc box-dependent-interacting protein |
| Ss_lncRNA_1969 | Antisense | intergenic | NDRG2-like |
| Ss_lncRNA_2198 | Antisense | intergenic | ethanolamine kinase 1-like |
| Ss_lncRNA_2753 | Sense | intergenic | unknown large open reading frame mRNA |
| Ss_lncRNA_4968 | Antisense | Intergenic | transcription factor Elf-1-like |
| Ss_lncRNA_4977 | Antisense | Promoter | TCR-alpha/delta V37-1 gene |

**Figure Legend**

**Supplementary figure 1: Venn diagram of differentially expressed lncRNAs by tissue and time.** The overlapping expression profiles of predicted long non-coding RNA transcripts is depicted in different colors across three tissues, gills (Green), liver (Blue), and head-kidney (Red).

**Supplementary table 1:** Primers sequence of lncRNAs used for RT-qPCR analysis

**Supplementary table 6.** Categories used to classify lncRNAs based on proximity to protein coding genes.
